# ScRNAPip: A systematic and dynamic pipeline for single‐cell RNA sequencing analysis

**DOI:** 10.1002/imt2.132

**Published:** 2023-08-31

**Authors:** Limin Xu, Jing Zhang, Yiqian He, Qianqian Yang, Tianhao Mu, Qiushi Guo, Yingqiang Li, Tian Tong, Shifu Chen, Richard D. Ye

**Affiliations:** ^1^ The Chinese University of Hong Kong Shenzhen Futian Biomedical Innovation R&D Center Shenzhen China; ^2^ School of Medicine The Chinese University of Hong Kong Shenzhen China; ^3^ HaploX Biotechnology Shenzhen China; ^4^ Kobilka Institute of Innovative Drug Discovery, School of Medicine The Chinese University of Hong Kong Shenzhen China

**Keywords:** bioinformatics, data analysis, single‐cell browsers, single‐cell RNA‐seq

## Abstract

With the advancement of sequencing technology, cell separation, and whole‐genome amplification techniques, single cell technology for genome sequencing is emerging gradually. In comparison to traditional genome sequencing at the multi‐cellular level, single‐cell sequencing can not only measure the gene expression level more accurately but also can detect a small amount of gene expression or rare noncoding RNA. This technology has garnered increasing interest among researchers engaged in single‐cell studies in recent years. Here, we developed a reproducible computational workflow for scRNA‐seq data analysis which including tasks like quality control, normalization, data correction, pseudotime analysis, copy number analysis, etc. We illustrate the application of these steps using publicly available datasets and provide practical recommendations for their implementation. This study serves as a comprehensive tutorial for researchers keen on single‐cell data analysis, aiding users in constructing and refining their own analysis pipelines.

## Highlights

- ScRNAPip is an analytic workflow used to analyze scRNA‐seq data.
- ScRNAPip integrates single‐cell report browsers, offering an interactive and intuitive graphic interface.
- Integrating copy number variation and genome instability, ScRNAPip enables quick evaluation of genomic alteration within single cell and aids in identifying tumor cells.

## Graphical abstract

ScRNAPip (https://github.com/OpenGene/scrnapip) is a reproducible computational workflow for scRNA‐seq data analysis. It incorporates multiple analysis modules including quality control, normalization, data correction, pseudotime analysis, copy number analysis, cellular interactions, and so on. This study will serve as a tutorial for researchers interested in single‐cell data analysis and help users build and update their analysis pipelines.

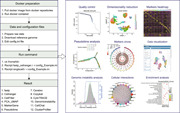

## INTRODUCTION

Single‐cell sequencing technology refers to a new technology for high‐throughput sequencing analysis of the genome, transcriptome, and epigenome at the level of a single cell. It can reveal the gene structure and gene expression status of a single cell and reflect the heterogeneity between cells. Owing to its high versatility, single‐cell omics technology plays an important role in the fields of cancer, microbiology, and neuroscience. The vast amount of new information gained through single‐cell RNA sequencing (scRNA‐seq) may reshape our understanding about gene regulation and cellular heterogeneity in disease [1].

Currently, more than 1500 tools have been developed for scRNA‐seq data analysis in over 30 categories [2]. This might be a great challenge for researchers in choosing appropriate tools for data analysis. In this study, we aim to establish a systematic, dynamic, and repeatable workflow and guide users through the key steps in the scRNA‐seq analysis, including data filtering, homogenization, dimensionality reduction analysis, difference analysis, pseudo‐time analysis, single‐cell browser, circos diagram, copy number variation (CNV), and genomic instability. This reproducible analysis pipeline can also be an easily modified configuration file to incorporate different data sources and will serve as a template to analyze and visualize single‐cell datasets in other disease models. In addition, considering the possibility of unexpected crashes after the program has been running for a long time, a restartable breakpoint is set in the process.

(Some single‐cell analysis workflows have already been developed, such as scAmpi https://github.com/ETH-NEXUS/scAmpi_single_cell_RNA) and scTyper (https://github.com/omicsCore/scTyper). Compared to these workflows, our workflow includes more analysis modules. For scTyper, (1) It does not include pseudotime analysis, cell communication, and functional analysis; (2) It does not have batch correction functionality; (3) It has fewer visualization results. For scampi, (1) It does not include tumor cell identification, cell communication, and pseudotime analysis. The disadvantages of ScRNAPip are: (1) Partial analysis only supports analysis using one software; (2) Weak applicability to species other than humans and mice.

The workflow is based on R [3] and Python, and uses software such as fastp (V0.23.2) [4, 5], CellRanger (V7.1.0) [6], Seurat (V4.3.0) [7], Monocle2 (V2.26.0) [8]. ScRNAPip does not support quality control and reads mapping of data from other platforms. However, if there is an expression matrix available, you can input it (following the format of 10X expression matrix) for other analysis points.

The pipeline currently supports both humans and mice. To analyze other species, please follow the steps below to create a reference genome and specify its location in the configuration file. (1) Download the reference genome's fa and gtf files from NCBI or Ensembl. Note that annotation files downloaded from NCBI are usually in gff format and need to be converted to gtf format using gffread. (2) Build the reference genome using the mkref command in CellRanger. The command is “CellRanger mkref –genome = genomename –fasta = genome.fa –genes = genome.gtf.” The reference genome constructed above can be used for CellRanger analysis to generate an expression matrix. In the subsequent advanced analysis, steps 1–6 and step 8 for cellular interactions can be run directly without any changes. Step 7 for copy number analysis, step 9 for Genomic instability analysis, and step 10 for pseudotime analysis only support humans and mice. Step 11 for enrichment analysis requires changing the database.

## METHOD

### The workflow of ScRNAPip

**Figure 1:**
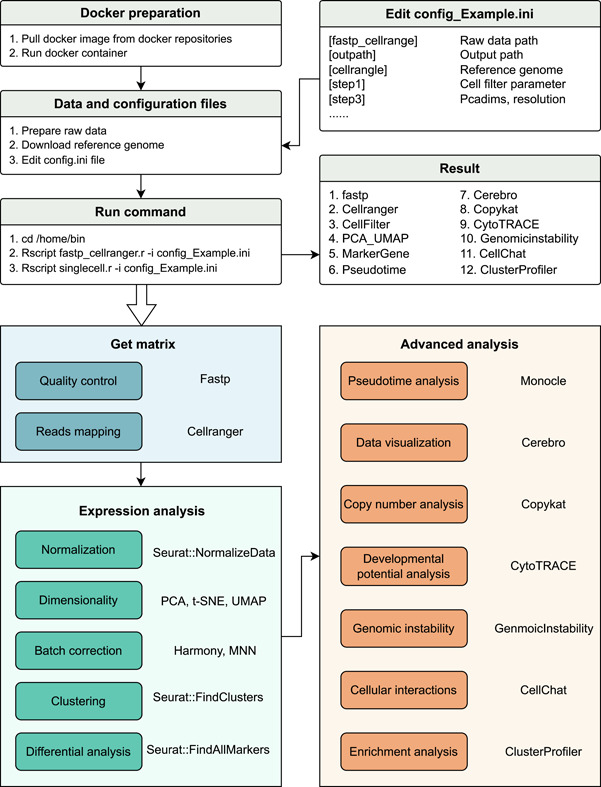
Workflow of ScRNAPip.

Generating single‐cell transcriptome data from a biological sample by ScRNAPip requires multiple steps (Figure 1). The tissue was dissociated into a single‐cell suspension using mechanical or enzymatic digestion. Qualified samples, gel beads, and oil were added to a 10X Genomics chip to generate water‐in‐oil structure system. The cells were then lysed and the gel beads were autolyzed to release massive primer sequences, which were reversely transcribed with the mRNA with PolyA to generate the first‐strand cDNA with 10X Barcode and Unique Molecular Identifier (UMI) information. After Gel Bead in Emulsion (GEM) was broken, first‐strand cDNA was purified by magnetic beads and PCR amplification was performed to obtain stable cDNA. The amplified cDNA was then fragmented for enzyme fragmentation and selected for appropriate length fragments. A 3′‐end transcriptome library containing P5 and P7 adaptors and the duplex index was constructed by end‐repairing, A‐tailing, and Read2 sequencing primers.

ScRNA‐seq analysis consists of several steps: (1) raw data filtering; (2) mapping to reference genome to generate expression matrix; (3) filtering multiple, apoptotic, and poor‐quality cells; (4) normalization; (5) dimensionality reduction and clustering; (6) differential analysis; (7) data visualization; (8) circos plot analysis; (9) copy number analysis; (10) developmental potential analysis; (11) genomic instability analysis; (12) cellular interactions; (13) enrichment analysis.

### Code availability

Install natively (macOS, Linux) from GitHub or docker using a docker container. If you are using Windows, you will need to install the necessary environment for Docker. The primary input and output files are listed.

The code used in this paper is publicly available in Docker (https://hub.docker.com/repository/docker/zhangjing12/scrnapip) or GitHub (https://github.com/OpenGene/scrnapip). Detailed practice tutorial can be found in the readme on GitHub or Docker.

The workflow integrates many module functions, such as filtering, clustering, clustering annotation, pseudotime, and there are many methods available. If you need to invoke them, just modify the configuration file (config.ini). Considering the unexpected program crashes after a long time running, restartable breakpoint also can be achieved by modifying the configuration file (Figure 1).

Minimum hardware requirements: 8‐core processor and 64 GB of RAM.

### Quality control

Captured transcript fragments that were processed by sequencers, termed “reads,” were stored in a text‐based format called FASTQ. There was a certain proportion of low‐quality data in raw data obtained by sequencing, which will cause a great interference with subsequent data analysis. Qualified sequencing data are a prerequisite for the reliability of subsequent data analysis results, and hence it is necessary to preprocess and evaluate the raw data. The 3′‐end scRNA‐seq data protocol for 10X requires specific analysis tools. Preprocessing the raw data using self‐developed open‐source software fastp to acquire the Clean Data for subsequent analysis. The filtering contents include the following parts: (1) cut read1 out to 28 bp; (2) the average quality of read2 is above 10; (3) remove read2 in which the N base has reached a certain percentage (default length of 5 bp). The parameters is “‐w 8 ‐l 28 ‐n 5 ‐q 10 ‐b 28 ‐B 0.” Both ‐l and ‐n can be adjusted through the parameters longr = 28 and ncode = 5 in the configuration file.

### Reads mapping

The pre‐processing analysis for the raw data of scRNA‐seq uses CellRanger that consists of four steps: (1) error correction of cell barcodes using predefined whitelists; (2) use STAR [9] to align the reads to a reference genome; (3) UMI correction and deduplication; (4) obtain gene expression data. It should be noted that although transcriptional reference can be used for comparison, it is preferable to use genome reference because it allows for easier removal of captured “off‐target” sequences. Eventually, each sample obtains gene expression matrices that can be further filtered and analyzed.

### Filtering low‐quality cells, doublets

In apoptotic cells, a high number of mitochondrial genes can often be detected. Most of these cells are not the “normal” cells we want. If too many or too few genes are detected, the cell may have problems with high probability. We identify multiple cells and cells with high mitochondrial content through filtration: (1) cells that have unique feature counts over 6000 or less than 200; (2) cells that have >20% mitochondrial gene expression. Different types of organizational cells have different filtering criteria, which can also be adjusted through the nFeature_RNA = [200, 6000] and percent_mt = [0, 20] settings in the configuration file. For doublets, we use DoubletFinder [10] for filtering. DoubletFinder identifies false‐negative Demuxlet classifications caused by doublets formed from cells with identical SNP profiles.

### Normalization

Normalization can improve the differences caused by uneven sequencing depth while retaining the real biological differences. BulkRNA often used the method of “size factor” [11, 12] (The size factor is the median ratio of the sample over a pseudosample, for each gene, the geometric mean of all samples), while there were a large number of zero‐expression genes in single cells, which was not suitable for single‐cell data. We use the SCTransform method in the Seurat to deal with the normalization problem. SCTransform uses regularized negative binomial regression to calculate the technical noise model, and the residual obtained is normalized value: (1) obtain highly variable genes, by default, 2000 highly variable genes will be returned; (2) after SCTransfrom normalization, the standard values are used for principal component analysis (PCA) [13], dimensionality reduction analysis, and difference analysis. In addition to SCTransform, we also provide Harmony [14] and MNN [15] for batch correction. In Tran HTN's study [16], the ASW index is used to evaluate performance. Seurat and Harmony are the best methods for balancing batch performance and cell type, with Harmony being the fastest in terms of computational speed, followed by MNN. The workflow supports multiple samples or datasets analysis, but batch effect correction is required.

### Dimensionality reduction and clustering

The scRNA‐seq data has high dimensionality, involving thousands of genes and a large number of cells. The purpose of dimensionality reduction is to preserve the key features of the data and project the high‐complexity data into a low‐dimensional space. First, PCA is used to summarize the genes of the first N principal components. Then, nonlinear dimensionality reduction methods were used, such as Uniform Manifold Approximation and Projection (UMAP) [17, 18] or t‐Distributed Stochastic Neighbor Embedding (t‐SNE) [19]. T‐SNE has limitations in slow computation time and large memory consumption. To reduce the dimensions of scRNA‐seq data, researchers developed UMAP and scvis. Compared to other methods, UMAP can provide the fastest running time, the highest reproducibility, and the most meaningful cell clusters. Therefore, UMAP is recommended, but we also offer the option of t‐SNE.

The goal of cell clustering is to group cells into clusters according to the similarity (or distance) of gene expression patterns in cells. These large groups become subgroups with mathematical significance. This is an important step and goal of scRNA‐seq data mining. The workflow uses a graph‐based clustering method: (1) draw a graph; (2) recognize the graph. Drawing usually consists of two steps, k‐nearest neighbor (KNN) and shared nearest neighbor (SNN). The recognition of graph is usually implemented by Louvain algorithm [20], which selects the group of cells with the highest similarity in the graph as a cell subgroup. The main advantages of Louvain algorithm are fastness and scalability. In addition, SLM algorithm, Leiden algorithm and other alternatives are provided.

For the generated cluster, singleR (V2.0.0) [21] is used to annotate. SingleR uses the known sequencing data of different types of cells as a reference, and then map the single‐cell data to references to obtain the approximate cell type. In addition, you can adjust the reference set for annotation by using the parameter singler = “yourref.rds” in the configuration file. The rds file can be obtained from the official website of singleR or generated according to the singleR tutorial. We recommend incorporating manual assistance into the annotation process after machine annotation as it yields more accurate results.

### Differential analysis

We use Findallmarker to find the differentially expressed genes in each cluster. By default, Seurat is used for analysis based on Wilcoxon rank sum test. Findmarker can also be used to analyze differences between two specific clusters, two cluster groups, or two sample groups. To obtain the list of differentially expressed genes, we take the selection criteria: (1) in the target subgroup or control subgroup, genes were expressed in more than 25% of cells; (2) corrected *p*‐value (P_val_adj) < 0.05; (3)｜logFC｜ > 0.5. Users can adjust the parameters findmarkers_testuse = “wilcox” and min_pct = 0.25 in the configuration file to make adjustments. The results of the difference analysis can be used for the next enrichment analysis. Users can specify the cluster or samples for difference analysis by editing the parameters in the configuration file. For example: difcluster.test.a = [0, 1], difcluster.test.b = [5, 6], difcluster.test.testuse = “wilcox” or difident.tVSn.a = [“sample1”], difident.tVSn.b = [“sample2”], difident.tVSn.testuse = “bimod”.

### Pseudotime analysis

Pseudotime analysis, also known as cell trajectory analysis, can infer the cell differentiation trajectory or the source of a certain type of cell differentiation during the development process. We used Monocle to perform pseudotime analysis of the cells. The pseudo‐locus construction process of cells includes the following steps: (1) choosing genes that define progress; (2) reducing the dimensionality of the data; (3) ordering the cells in pseudotime.

### Data visualization

We used Cerebro (V1.2.2) [22] to visualize single‐cell data, which is suitable for those who are not familiar with bioinformatics analysis to perform data exploration. Cerebro's functions mainly include: (1) displaying interactive dimensionality reduction results; (2) displaying differentially expressed genes; (3) exporting pictures and data. In addition, the software provides 2D and 3D displays of UMAP, which can be downloaded at the following website (https://github.com/cerebroapp/cerebro) and supports both the Windows and Linux system. If users have experience in RStudio, the R package cerebroApp can be used to explore scRNA‐seq data.

### Circos plot analysis

To display the marker gene more intuitively, circos (V0.4.15) [23] plot is used. Circos diagram shows the marker genes of all clusters or cell types, draws bubble, heatmap, and scatter diagrams [24] on different tracks. Bubble chart: different samples are distinguished by color. The size of the dot represents the proportion of cells expressing the marker gene in each cluster or sample. Select one marker gene for each cluster or type to display. Heatmap: color represents the gene expression level. Scatter map: the ordinate indicates the size of marker gene log fold change, and the red points indicate significance at *p*‐value < 0.05. Mark the marker gene selected by each cluster or type in the scatter diagrams.

### Copy number analysis

CNV, also known as copy number polymorphism (CNP), refers to the variation of DNA fragments ranging in size from 1 kb to 3 Mb that result from genomic rearrangements. CopyKAT [25] uses a comprehensive Bayesian method to analyze single‐cell transcriptome data and identify whole‐genome CNVs with a resolution of 5 MB in single cells, enabling the differentiation of tumor cells from normal cells and tumor subclones for further in‐depth analysis. Therefore, this tool can be widely used in the study of various solid tumors.

### Developmental potential analysis

Previous studies have found that stem cells express more genes than mature cell types. CytoTRACE [26] scores cells based on the number of detectable expressed genes (gene count) to evaluate the differentiation potential of different single‐cell subpopulations. This approach can overcome the dependence on prior knowledge of developmental direction or intermediate states and independently and more robustly identify the developmental potential of cells. This result helps us identify cell types, construct cell development trajectories, and combining it with Monocle2 analysis results can help determine the start and end stages of the trajectory.

### Genomic instability analysis

The genomicInstability package is a software package used for genomic instability analysis from single‐cell RNA sequencing data. It uses the aREA algorithm to quantify the enrichment of contiguous gene sets on the gene expression profile, quantitatively estimate the correlation between gene expression and chromosome position (loci‐blocks) on single‐cell gene expression profiles, and estimate the genomic instability score for each cell.

### Cellular interactions

The cell–cell communication is extremely complex and is generally mediated by ligands such as hormones, growth factors, chemokines, cytokines, and neurotransmitters, facilitating the exchange of information between cells. CellChat [27] is a tool that quantitatively infers and analyzes cell–cell communication networks from scRNA‐seq data. It models the communication between cells A and B by utilizing prior knowledge of ligand‐receptor interactions based on currently known/validated pairs, and predicts their communication status. This approach allows CellChat to construct a comprehensive cell‐cell communication network. Additionally, CellChat provides numerous functions for further data exploration, analysis, and visualization.

### Enrichment analysis

Enrichment refers to the process of classifying genes based on prior knowledge (genome annotation information). Through enrichment analysis, we can categorize differentially expressed genes or substances based on their functions, and correlate functions with phenotypes. In this process, we use clusterProfiler [28] for functional enrichment analysis (which is based on the principle of hypergeometric distribution). The main functional databases used in this process include GO, KEGG, Reactome, and so on.

## RESULT

In addition, we downloaded and tested four previously published datasets for the demonstration: GSM3564834, GSM3564835, GSM3564836, and GSM3564837 [29] (https://www.ncbi.nlm.nih.gov/Traces/study/?acc=PRJNA515497&o=acc_s%3Aa). We provided a table based on our analysis experience for users to reference and estimate the runtime (based on 16 cores and 64 GB RAM) (Table 1).

**Table 1:** Running time.

Sample number	Data size of sample	Total disk memory	Running time (h)
1	110G	200G	12
4	110G	550G	36
8	110G	1T	80

*Note*: Different hardware configurations may also result in differences in analysis time. Total disk memory includes raw data.

**Figure 2:**
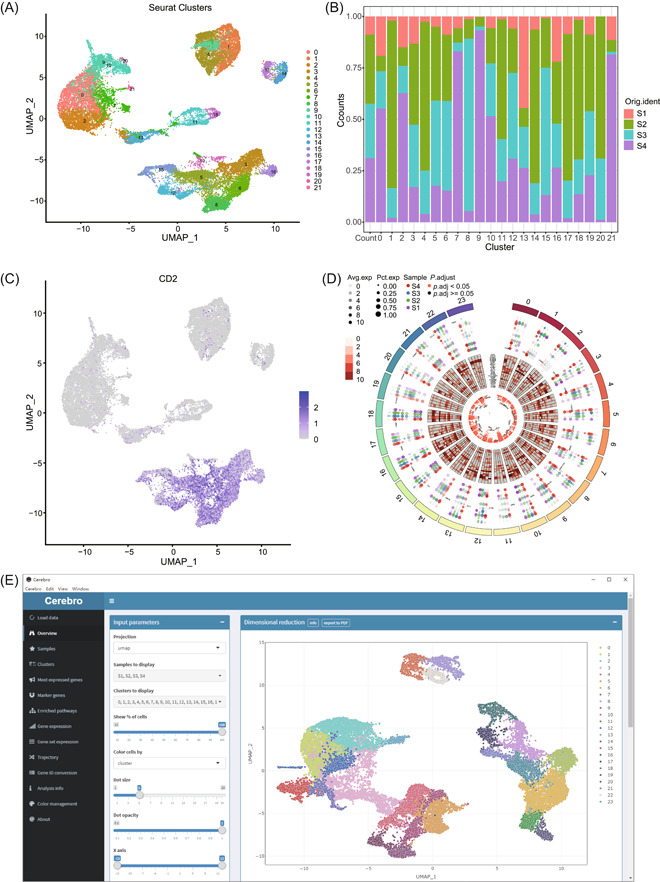
Example of the main outputs of the workflow. (A) Uniform Manifold Approximation and Projection (UMAP) plot displaying cell clustering in single‐cell data. (B) Bar plot showing the proportions of different clusters in each sample. (C) Expression levels of the CD2 marker gene in individual cells. (D) Circos plot displaying marker genes for all clusters or cell types, with bubble plots, heatmaps, and scatter plots arranged on different tracks. (E) This is a screenshot of the Cerebro software interface, used to display single‐cell data analysis workflows or visualization tools.

**Figure 3:**
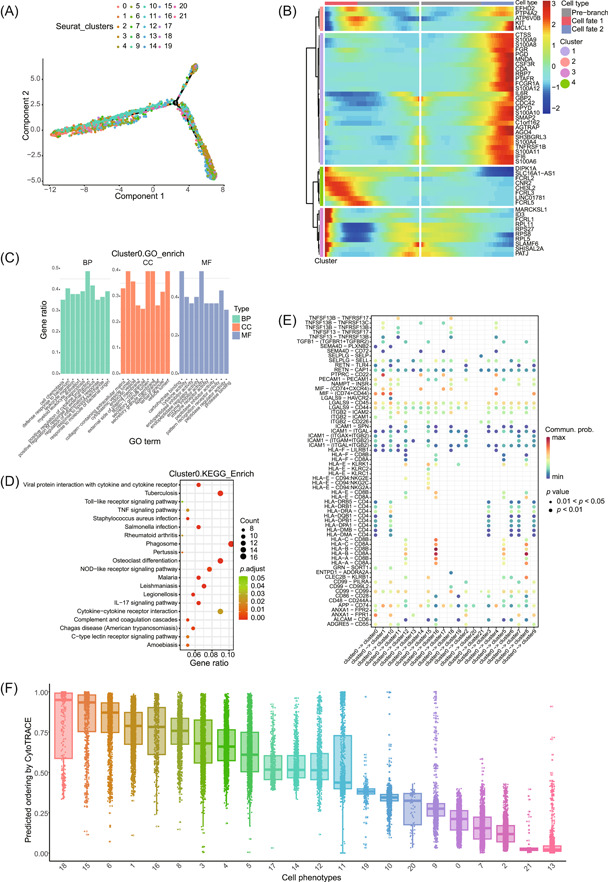
Example of the main outputs of the workflow. (A) Cell trajectory constructed using Monocle. (B) The heatmap grouped the differentially expressed genes from the branched expression analysis modeling (beam) into four classes, displaying the fate‐determining genes associated with prebranch differentiation into cell fate 1 and cell fate 2. (C) The bar plot displays significant enrichment of gene ontology terms. (D) The bubble plot illustrates significantly enriched KEGG pathways. The size of each dot represents the number of significantly enriched genes in the pathway, and the color represents the adjusted *p*‐value. (E) All the significant ligand‐receptor pairs with cluster 0 as the ligand. The dot color and size represent the calculated communication probability and *p*‐values, respectively. (F) All cells were scored using cytoTRACE (Cellular Trajectory Reconstruction Analysis using gene Counts and Expression), box plot shows the developmental potential of different clusters.

Partial results are shown in Figures 2 and 3.

## CONCLUSIONS AND OUTLOOK

The tutorial was designed to establish a repeatable and efficient single‐cell RNA analysis pipeline. Hopefully, this pipeline will provide a suitable entry point into this field for newcomers and contribute to the establishment of the Human Cell Atlas in scRNA‐seq. ScRNA‐seq is a rapidly developing field, and developers continue to propose new methods. New analytical tools may be available in the future to further expand the application of scRNA‐seq. The software used in the workflow (Seurat, scanpy, R) will also be updated continuously to improve our pipeline.

There are now many bioinformatics analysis workflows based on Docker packaging. Overall, this new approach provides a beginner‐friendly and versatile solution with various configurations. However, users need to confirm its suitability and make adjustments and additions based on their specific requirements before using it.

Its advantages are: (1) simplified installation, no need to install various bioinformatics software. (2) beginner‐friendly, analysis operations can be completed with just one click. (3) comprehensive, covers the entire process from raw sequencing data to personalized analysis. (4) flexibility, allows users to customize adjustments based on their research questions.

Disadvantages: (1) limited to certain species: strong applicability to human and mouse samples, weaker adaptability to other species. (2) dependency on prior knowledge, although beginners can quickly get started, some background knowledge is still required. (3) computational resources, this workflow is applied on a server, so users should evaluate their computational resources and storage capacity to ensure they can meet the memory requirements of the workflow.

Single‐cell multiomics integrates multiple technologies such as single‐cell sequencing, mass spectrometry analysis, and fluorescence microscopy, to simultaneously obtain multiple omics information of cells, including the genome, transcriptome, proteome, and metabolome. It helps us to gain a deeper understanding of the function and characteristics of individual cells, uncover cell heterogeneity, and discover new cell subtypes and cell state transitions. However, single‐cell multiomics research also faces challenges such as sample preparation, complex experimental procedures, and the interpretation of multiple omics data. In summary, single‐cell multi‐omics provides us with a new way to study cells and is expected to play an important role in disease research and personalized medicine.

In recent years, artificial intelligence technologies such as deep learning have achieved remarkable achievements in image recognition, natural language processing, and other fields. AI technologies have made significant impacts on multiple fields of biological information. With the rapid development of artificial intelligence, there may be significant breakthroughs in AI‐based single‐cell RNA sequencing analysis. However, applying deep learning to single‐cell sequencing data analysis faces many challenges, including data sparsity, dimensionality catastrophe, and difficulty in interpretation. To achieve this goal, we need to create better analytical tools and methods and continue to work together to achieve a stronger and repeatable solution.

## AUTHOR CONTRIBUTIONS

Limin Xu, Jing Zhang, Yingqiang Li, and Tian Tong developed this tool and wrote the manuscript. Yiqian He, Qianqian Yang, Tianhao Mu, and Qiushi Guo conducted the software testing and manuscript polishing. Shifu Chen and Richard D. Ye supervised this project.

## CONFLICT OF INTEREST STATEMENT

The authors declare no conflict of interest.

## Data Availability

The presented tool can be found at https://github.com/OpenGene/scrnapip. The code scripts used for data processing, analysis, and visualization have been deposited at https://github.com/OpenGene/scrnapip/tree/main/docker/data. Supplementary materials (scripts, graphical abstract, slides, videos, Chinese translated version and update materials) may be found in the online DOI or iMeta Science http://www.imeta.science/. The data that support the findings of this study are available in GEO (https://www.ncbi.nlm.nih.gov/Traces/study/?acc=PRJNA515497&o=acc_s%3Aa): GSM3564834 GSM3564835 GSM3564836 GSM3564837.

## References

[1] Griffiths, Jonathan A. , Antonio Scialdone , and John C. Marioni . 2018. “Using Single‐Cell Genomics to Understand Developmental Processes and Cell Fate Decisions.” Molecular Systems Biology 14: e8046. 10.15252/msb.20178046 29661792 PMC5900446

[2] scRNA‐tools. Accessed June 1, 2023. https://www.scrna-tools.org/

[3] R Core Team . 2022. R: A language and environment for statistical computing. R Foundation for Statistical Computing.

[4] Chen, Shifu . 2023. “Ultrafast One‐Pass FASTQ Data Preprocessing, Quality Control, and Deduplication Using Fastp.” Imeta 2: e107. 10.1002/imt2.107 PMC10989850 38868435

[5] Chen, Shifu , Yanqing Zhou , Yaru Chen , and Jia Gu . 2018. “Fastp: An Ultra‐Fast All‐in‐One FASTQ Preprocessor.” Bioinformatics 34: i884–i890. 10.1093/bioinformatics/bty560 30423086 PMC6129281

[6] Zheng, Grace X. Y. , Jessica M. Terry , Phillip Belgrader , Paul Ryvkin , Zachary W. Bent , Ryan Wilson , Solongo B. Ziraldo , et al. 2017. “Massively Parallel Digital Transcriptional Profiling of Single Cells.” Nature Communications 8: 14049. 10.1038/ncomms14049 PMC5241818 28091601

[7] Hao, Yuhan , Stephanie Hao , Erica Andersen‐Nissen , William M. Mauck , Shiwei Zheng , Andrew Butler , Maddie J. Lee , et al. 2021. “Integrated Analysis of Multimodal Single‐Cell Data.” Cell 184: 3573–3587. 10.1016/j.cell.2021.04.048 34062119 PMC8238499

[8] Qiu, Xiaojie , Andrew Hill , Jonathan Packer , Dejun Lin , Yi‐An Ma , and Cole Trapnell . 2017. “Single‐Cell mRNA Quantification and Differential Analysis with Census.” Nature Methods 14: 309–315. 10.1038/nmeth.4150 28114287 PMC5330805

[9] Dobin, Alexander , Carrie A. Davis , Felix Schlesinger , Jorg Drenkow , Chris Zaleski , Sonali Jha , Philippe Batut , Mark Chaisson , and Thomas R. Gingeras . 2013. “STAR: Ultrafast Universal RNA‐seq Aligner.” Bioinformatics 29: 15–21. 10.1093/bioinformatics/bts635 23104886 PMC3530905

[10] McGinnis, Christopher S. , Lyndsay M. Murrow , and Zev J. Gartner . 2019. “DoubletFinder: Doublet Detection in Single‐Cell RNA Sequencing Data Using Artificial Nearest Neighbors.” Cell Systems 8: 329–337. 10.1016/j.cels.2019.03.003 30954475 PMC6853612

[11] Anders, Simon , and Wolfgang Huber . 2010. “Differential Expression Analysis for Sequence Count Data.” Genome Biology 11: R106. 10.1186/gb-2010-11-10-r106 20979621 PMC3218662

[12] Love, Michael I. , Wolfgang Huber , and Simon Anders . 2014. “Moderated Estimation of Fold Change and Dispersion for RNA‐seq Data With DESeq. 2.” Genome Biology 15: 550. 10.1186/s13059-014-0550-8 25516281 PMC4302049

[13] Lever, Jake , Martin Krzywinski , and Naomi Altman . 2017. “Principal Component Analysis.” Nature Methods 14: 641–642. 10.1038/nmeth.4346

[14] Korsunsky, Ilya , Nghia Millard , Jean Fan , Kamil Slowikowski , Fan Zhang , Kevin Wei , Yuriy Baglaenko , et al. 2019. “Fast, Sensitive and Accurate Integration of Single‐Cell Data With Harmony.” Nature Methods 16: 1289–1296. 10.1038/s41592-019-0619-0 31740819 PMC6884693

[15] Haghverdi, Laleh , Aaron T. L. Lun , Michael D. Morgan , and John C. Marioni . 2018. “Batch Effects in Single‐Cell RNA‐Sequencing Data Are Corrected By Matching Mutual Nearest Neighbors.” Nature Biotechnology 36: 421–427. 10.1038/nbt.4091 PMC6152897 29608177

[16] Tran, Hoa Thi Nhu , Kok Siong Ang , Marion Chevrier , Xiaomeng Zhang , Nicole Yee Shin Lee , Michelle Goh , and Jinmiao Chen . 2020. “A Benchmark of Batch‐Effect Correction Methods for Single‐Cell RNA Sequencing Data.” Genome Biology 21: 12. 10.1186/s13059-019-1850-9 31948481 PMC6964114

[17] Becht, Etienne , Leland McInnes , John Healy , Charles‐Antoine Dutertre , Immanuel W. H. Kwok , Lai Guan Ng , Florent Ginhoux , and Evan W. Newell . 2019. “Dimensionality Reduction for Visualizing Single‐Cell Data Using UMAP.” Nature Biotechnology 37: 38–44. 10.1038/nbt.4314 30531897

[18] McInnes, Leland , John Healy , Nathaniel Saul , and Lukas Großberger . 2018. “UMAP: Uniform Manifold Approximation and Projection.” Journal of Open Source Software 3: 861. 10.21105/joss.00861

[19] Laurens, Van Der Maaten , and Geoffrey Hinton . 2008. “Visualizing Data Using t‐SNE.” Journal of Machine Learning Research 9: 2579–2605. https://jmlr.org/papers/v9/vandermaaten08a.html

[20] Blondel, Vincent D. , Jean‐Loup Guillaume , Renaud Lambiotte , and Etienne Lefebvre . 2008. “Fast Unfolding of Communities in Large Networks.” Journal of Statistical Mechanics: Theory and Experiment 2008: P10008. 10.1088/1742-5468/2008/10/P10008

[21] Aran, Dvir , Agnieszka P. Looney , Leqian Liu , Esther Wu , Valerie Fong , Austin Hsu , Suzanna Chak , et al. 2019. “Reference‐Based Analysis of Lung Single‐Cell Sequencing Reveals a Transitional Profibrotic Macrophage.” Nature Immunology 20: 163–172. 10.1038/s41590-018-0276-y 30643263 PMC6340744

[22] Hillje, Roman , Pier Giuseppe Pelicci , and Lucilla Luzi . 2020. “Cerebro: Interactive Visualization of scRNA‐seq Data.” Bioinformatics 36: 2311–2313. 10.1093/bioinformatics/btz877 31764967 PMC7141853

[23] Gu, Zuguang , Lei Gu , Roland Eils , Matthias Schlesner , and Benedikt Brors . 2014. “Circlize Implements and Enhances Circular Visualization in R.” Bioinformatics 30: 2811–2812. 10.1093/bioinformatics/btu393 24930139

[24] Wickham, Hadley . 2009. *Ggplot2: Elegant Graphics for Data Analysis*. 10.1007/978-0-387-98141-3

[25] Gao, Ruli , Shanshan Bai , Ying C. Henderson , Yiyun Lin , Aislyn Schalck , Yun Yan , Tapsi Kumar , et al. 2021. “Delineating Copy Number and Clonal Substructure in Human Tumors From Single‐Cell Transcriptomes.” Nature Biotechnology 39: 599–608. 10.1038/s41587-020-00795-2 PMC8122019 33462507

[26] Gulati, Gunsagar S. , Shaheen S. Sikandar , Daniel J. Wesche , Anoop Manjunath , Anjan Bharadwaj , Mark J. Berger , Francisco Ilagan , et al. 2020. “Single‐Cell Transcriptional Diversity is a Hallmark of Developmental Potential.” Science 367: 405–411. 10.1126/science.aax0249 31974247 PMC7694873

[27] Jin, Suoqin , Christian F. Guerrero‐Juarez , Lihua Zhang , Ivan Chang , Raul Ramos , Chen‐Hsiang Kuan , Peggy Myung , Maksim V. Plikus , and Qing Nie . 2021. “Inference and Analysis of Cell‐Cell Communication Using Cel lChat.” Nature Communications 12: 1088. 10.1038/s41467-021-21246-9 PMC7889871 33597522

[28] Yu, Guangchuang , Li‐Gen Wang , Yanyan Han , and Qing‐Yu He . 2012. “Clusterprofiler: An R Package for Comparing Biological Themes Among Gene Clusters.” OMICS: A Journal of Integrative Biology 16: 284–287. 10.1089/omi.2011.0118 22455463 PMC3339379

[29] Zhao, Juanjuan , Shuye Zhang , Yang Liu , Xiaomeng He , Mengmeng Qu , Gang Xu , Hongbo Wang , et al. 2020. “Single‐Cell RNA Sequencing Reveals the Heterogeneity of Liver‐Resident Immune Cells in Human.” Cell Discovery 6: 22. 10.1038/s41421-020-0157-z 32351704 PMC7186229

